# Exploring Liraglutide in Lithium–Pilocarpine-Induced Temporal Lobe Epilepsy Model in Rats: Impact on Inflammation, Mitochondrial Function, and Behavior

**DOI:** 10.3390/biomedicines12102205

**Published:** 2024-09-27

**Authors:** Fatma Merve Antmen, Zeynep Fedaioglu, Dilan Acar, Ahmed Kerem Sayar, Ilayda Esma Yavuz, Ece Ada, Bengisu Karakose, Lale Rzayeva, Sevcan Demircan, Farah Kardouh, Simge Senay, Meltem Kolgazi, Guldal Suyen, Devrim Oz-Arslan

**Affiliations:** 1 Department of Physiology, Graduate School of Health Sciences, Acibadem Mehmet Ali Aydinlar University, Istanbul 34752, Türkiye; merve.antmen@acibadem.edu.tr (F.M.A.);; 2 Biobank Unit, Acibadem Mehmet Ali Aydinlar University, Istanbul 34752, Türkiye; 3 Department of Molecular Biology and Genetics, Faculty of Arts and Sciences, Acibadem Mehmet Ali Aydinlar University, Istanbul 34752, Türkiye; 4 School of Medicine, Acibadem Mehmet Ali Aydinlar University, Istanbul 34752, Türkiye; 5 Department of Medical Biotechnology, Graduate School of Health Sciences, Acibadem Mehmet Ali Aydinlar University, Istanbul 34752, Türkiye; 6 Department of Physiology, School of Medicine, Acibadem Mehmet Ali Aydinlar University, Istanbul 34752, Türkiye; 7 Department of Biophysics, School of Medicine, Acibadem Mehmet Ali Aydinlar University, Istanbul 34752, Türkiye

**Keywords:** epilepsy, liraglutide, antioxidant pathways, inflammation, mitochondria, behavior

## Abstract

**Background/Objectives**: Glucagon-like peptide-1 receptor agonists such as liraglutide are known for their neuroprotective effects in neurodegenerative disorders, but their role in temporal lobe epilepsy (TLE) remains unclear. We aimed to investigate the effects of liraglutide on several biological processes, including inflammation, antioxidant defense mechanisms, mitochondrial dynamics, and function, as well as cognitive and behavioral changes in the TLE model. **Methods**: Low-dose, repeated intraperitoneal injections of lithium chloride–pilocarpine hydrochloride were used to induce status epilepticus (SE) in order to develop TLE in rats. Fifty-six male Sprague Dawley rats were subjected and allocated to the groups. The effects of liraglutide on inflammatory markers (NLRP3, Caspase-1, and IL-1β), antioxidant pathways (Nrf-2 and p-Nrf-2), and mitochondrial dynamics proteins (Pink1, Mfn2, and Drp1) were evaluated in hippocampal tissues via a Western blot. Mitochondrial function in peripheral blood mononuclear cells (PBMCs) was examined using flow cytometry. Cognitive-behavioral outcomes were assessed using the open-field, elevated plus maze, and Morris water maze tests. **Results**: Our results showed that liraglutide modulates NLRP3-mediated inflammation, reduces oxidative stress, and triggers antioxidative pathways through Nrf2 in SE-induced rats. Moreover, liraglutide treatment restored Pink1, Mfn2, and Drp1 levels in SE-induced rats. Liraglutide treatment also altered the mitochondrial function of PBMCs in both healthy and epileptic rats. This suggests that treatment can modulate mitochondrial dynamics and functions in the brain and periphery. Furthermore, in the behavioral aspect, liraglutide reversed the movement-enhancing effect of epilepsy. **Conclusions**: This research underscores the potential of GLP-1RAs as a possibly promising therapeutic strategy for TLE.

## 1. Introduction

Epilepsy is a common neurological disorder that affects more than 70 million people around the world [1,2]. Epilepsy is typically associated with an elevated risk of developing other physiological and psychological comorbidities, including depression and anxiety [3]. The current therapies for epilepsy are based only on suppressing seizure development [4]. A substantial proportion of patients with epilepsy, approximately one-third, are refractory to pharmacological treatment, with temporal lobe epilepsy (TLE) representing the most prevalent form of drug-resistant epilepsy [5,6]. In TLE, seizures originate from the temporal lobe of the brain, and one of the main areas of damage is the hippocampus [7].

There is growing evidence that links mitochondrial dysfunction, neuroinflammation, excitotoxicity, and oxidative stress to the causes of neurodegenerative diseases, including epilepsy [8,9]. Although mitochondrial malfunction and neuroinflammation lead to neuronal cell death, the mechanisms underlying neurodegeneration remain poorly understood. Despite extensive research, the pathogenesis of TLE still is not fully understood. However, there is compelling evidence indicating that mitochondrial dysfunction plays a pivotal role in the pathogenesis of TLE [9]. Reduced adenosine triphosphate (ATP) levels, alterations in neuronal calcium homeostasis, and oxidative protein degradation via reactive oxygen species (ROS) contribute to an imbalance between neural network excitation and inhibition, which may serve as direct mechanisms linking mitochondrial dysfunction to TLE [8,10]. Studies have demonstrated that oxidative stress causes mitochondrial fission in neurons, whereas mitofusin 2 (Mfn2), which is responsible for mitochondrial fusion, plays protective roles during neuronal injury factor [11,12]. When damaged mitochondria cannot be properly removed through mitophagy, a type of selective autophagy, several stress factors are released into the cytoplasm and extracellular environment, accelerating the neuroinflammatory process. Furthermore, the nucleotide-binding oligomerization domain-like receptor pyrin domain containing 3 (NLRP3) inflammasome is activated through mitochondria-derived ROS. Recent research on refractory TLE has shown that NLRP3 may contribute to disease development and that the inhibition of NLRP3 expression may reduce local brain damage by reducing interleukin-1β (IL-1β) expression [13]. Furthermore, the NLRP3 inflammasome pathway represents a potential target for antiepileptogenic treatment strategies. Nuclear factor erythroid 2-related factor 2 (Nrf2), a transcription factor, has been identified as a regulator protein responsible for NLRP3 inflammasome activation [14]. Nrf2 is essential for controlling a wide range of genes involved in antioxidant defense and detoxification processes [15]. It is activated via ROS, and once activated, it protects cells against inflammation-induced oxidative stress [16]. In addition to the evidence indicating neuroinflammation, there is also a body of research suggesting mitochondrial dysfunction in peripheral blood cells in neurological disorders, including autism spectrum disorders, Parkinson’s disease, and Alzheimer’s disease [17,18,19]. Moreover, some recent studies have highlighted that changes in immune-system cells may be a critical component of the initial seizure-inducing event, suggesting immune modulation as a potential therapeutic option in epilepsy. However, how the mitochondrial function of peripheral blood mononuclear cells (PBMCs) changes in epilepsy remains elusive. Therefore, one promising strategy for developing therapeutic strategies for TLE may be the use of drugs to reduce neuroinflammation, oxidative stress, and mitochondrial dysfunction.

Glucagon-like peptide-1 (GLP-1) agonists, such as liraglutide, are commonly used to manage type 2 diabetes mellitus (T2DM), and there is evidence suggesting that they also offer therapeutic benefits for various neurodegenerative diseases. GLP-1 receptors (GLP-1Rs) are widely distributed in the brain, including the hippocampus [13,20,21,22,23]. Liraglutide has been shown to have anticonvulsant and neuroprotective properties in the context of epilepsy by reducing oxidative stress in pentylenetetrazol (PTZ)-induced seizures [24]. Furthermore, liraglutide treatment showed antiepileptogenic and neuroprotective effects in a unilateral kainic acid (KA) model of TLE [25]. A recent study reported that it affects mitochondrial dynamics in a model of Parkinson’s disease [26]. However, its effects on neuroinflammation, oxidative stress, and mitochondrial function during epileptogenesis remain unclear.

The majority of knowledge of TLE comes from status epilepticus (SE) induction. The lithium–pilocarpine combination has been the most commonly used method for inducing SE for the development of TLE. The main characteristics of TLE can be reproduced in rats via systemic injections of lithium and pilocarpine, allowing for a reliable animal model for testing and development purposes. In this model of TLE rats; following SE, a latent period occurs, which is followed by spontaneous recurrent seizures (SRSs) [27]. In this study, we employed a low-dose, repeated injection of lithium–pilocarpine to induce SE and subsequently TLE in rats, resulting in a relatively low mortality rate [28]. Afterwards, we examined whether liraglutide has any neuroprotective effects on SE-induced TLE and its potential impacts on neuroinflammation, mitochondrial dynamics, and cognitive and behavioral functions in rats.

## 2. Materials and Methods

### 2.1. Animals

A total of fifty-six young male Sprague Dawley rats, with a weight range of 200–300 g, were obtained from the Acibadem University Laboratory Animal Research Center (ACUDEHAM). The rats were acclimated to the laboratory environment for 7 days prior to the start of the experiment. We also implemented a handling protocol for the rats to facilitate familiarization and minimize external stress. This was achieved by handling laboratory rats for a duration of 5 min once daily for a consecutive 5-day period. They were housed at room temperature (24 °C ± 1 °C) with a 12-h light–dark cycle (lights on at 7:00 a.m.) and had unrestricted access to food and water. Every effort was made to reduce the number of animals used and to minimize animal suffering. Given the inherently challenging, prolonged, and complex nature of epilepsy studies, following SE, the rats exhibited difficulties with feeding and water intake. Consequently, fluid intake challenges were addressed by providing a necessary replacement, while feeding difficulties were resolved by soaking and placing the animals’ food in their cages. Moreover, to prevent any potential irritation or injury to the animals, each SE-induced rat was placed in a separate cage during this period. All procedures related to animal husbandry, experimental protocols, and euthanasia were approved by the Acibadem University Experimental Animal Research Ethics Committee in Istanbul, Turkey (approval number: HDK-2021-28). All procedures followed the Animal Research: Reporting of In Vivo Experiments guideline and were conducted in accordance with EU Directive 2010/63/EU on animal experiments.

### 2.2. Chemicals and Drugs

The following compounds were purchased from Sigma-Aldrich^®^ (St. Louis, MO, USA): lithium chloride, methyl scopolamine, and pilocarpine hydrochloride. Thiopental sodium was procured from Ibrahim Etem of the Menarini Group in Turkey. Liraglutide (commercialized as Victoza^®^) was purchased from Novo Nordisk (Rome, Italy) in the form of a prefilled pen containing 18 mg of liraglutide in 3 mL. Lithium chloride (3 mEq/kg) was dissolved in water for injection and administered intraperitoneally (IP). Methyl scopolamine (1 mg/kg), pilocarpine hydrochloride (20 mg/kg), and liraglutide (300 µg/kg) were dissolved in 0.9% saline and administered IP. Thiopental sodium (30 mg/kg) was dissolved in water for injection and administered IP. The applications of all chemicals and drugs were calculated to be given in approximately 0.1 mL, according to each animal’s weight. The doses of the drugs employed were selected based on the findings of previous studies [25,28,29].

### 2.3. Study Design

**Figure 1:**
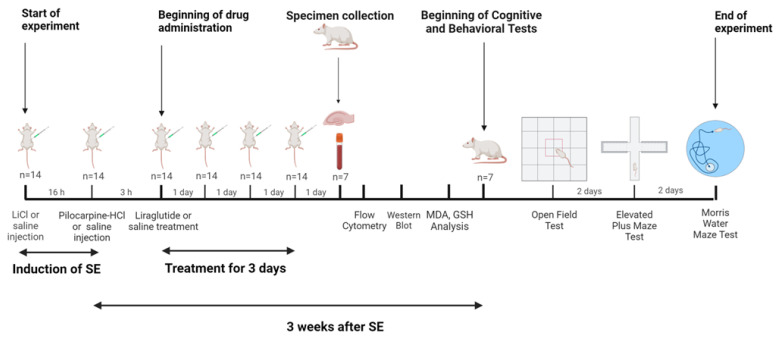
Timeline and experimental design (created using BioRender.com).

Four experimental groups were set up, and treatments were given 3 h after the start of SE to target the early stage of epileptogenesis. The first group served as the control (C) group (n = 14) and received IP injections of 0.9% saline (1 mg/kg) at times consistent with the SE groups. The second group was the liraglutide control group (LIR) (n = 14). They received IP injections of 0.9% saline (1 mg/kg) and liraglutide (300 µg/kg; 300 µg/1 mL) for 3 days, at times compatible with the SE groups. The third group, SE-induced (E+SAL) (n = 14), received IP injections of 0.9% saline (1 mg/kg) 3 h after SE onset for 3 days. The last group was the SE-induced and liraglutide-treated group (E+LIR) (n = 14). The final group, SE-induced with liraglutide treatment (E+LIR) (n = 14), was administered lithium–pilocarpine to induce SE, followed by IP injections of liraglutide (300 µg/kg; 300 µg/1 mL) 3 h post-SE onset for 3 days. After the 3-day treatment, seven rats from each group were euthanized to study changes in mitochondrial dynamics, inflammation, and antioxidant capacity. First, blood samples from the rats (n = 7 for each group) were obtained via cardiac puncture under low-level anesthesia with isoflurane (1–2% oxygen). These anesthetic conditions were maintained throughout the process. The collected blood samples were immediately processed for flow cytometry. Following blood collection, the same animals were used for hippocampus isolation to conduct Western blot analysis. For this purpose, isoflurane level was increased to 4–5% in order to induce deep anesthesia in rats, which was confirmed by the absence of a response to a toe pinch, followed by rapid decapitation. The brains were removed, and then the dissected hippocampal tissues were immediately frozen in liquid nitrogen and stored at −80 °C until protein extraction. Lastly, the remaining seven rats from each group were subjected to cognitive-behavioral tasks, including the Morris water maze, the elevated plus maze, and open-field tests, three weeks following the onset of SE. The experimental design was depicted in Figure 1.

### 2.4. Lithium–Pilocarpine-Model of TLE

A low-dose, repetitive lithium–pilocarpine model was employed to induce SE [28]. In brief terms, the rats were given an IP injection of 3 mEq/kg lithium chloride, followed 16–20 h later via an IP injection of pilocarpine hydrochloride (20 mg/kg). Pilocarpine was administered every 30 min until SE was induced, up to a maximum of five doses. To mitigate peripheral cholinergic effects, methyl scopolamine (1 mg/kg, IP) was given 30 min before the first pilocarpine dose. To reduce mortality, thiopental sodium (30 mg/kg, IP) was administered 90 min after SE onset, with a second dose given 10 min later if needed. The rats were then observed for 1 h to monitor SE development and assess behavioral seizure stages. Seizures were scored according to a modified version of Racine’s scale (1972), adapted by Wamil et al. (1989): stage 1 included akinesia, staring, and salivation; stage 2 involved head and forelimb clonus with wet dog shakes; stage 3 was characterized by rearing with forelimb clonus; stage 4 involved rearing and falling with generalized convulsions and status epilepticus; and stage 5 resulted in death [30,31].

### 2.5. Flow Cytometry

Mitochondrial function in PBMCs from all experimental groups was assessed using flow cytometry with mitochondrial-specific fluorescent dyes. A total of 28 rats (n = 7 for each group) were used for this experiment. First, PBMCs were isolated from blood samples via density-gradient centrifugation using Histopaque^®^-1077 (Sigma-Aldrich^®^, St. Louis, MO, USA), as described previously [32]. The isolated PBMCs were centrifuged at 500× *g* for 5 min, followed by washing with 1 × phosphate-buffered saline (PBS). Cells were immediately used for flow cytometric measurement, and trypan blue staining was performed for live and dead cell counts. Approximately 2 × 10^5^ cells from each group were then stained with 100 nM MitoTracker Red CMXRos, 5 μM MitoSOX Red, and 50 nM MitoTracker Green FM (Thermo-Fisher Scientific, Waltham, MA, USA) for 20 min at 37 °C to assess mitochondrial membrane potential, superoxide production, and mitochondrial mass, respectively. After staining, the cells were washed with 1× PBS, resuspended in 400 μL of PBS, and transferred to flow cytometry tubes for analysis using a BD FACSVerse system. A total of 10,000 events were collected, properly gated, and analyzed with BD FACSuite software (version number: v1.0.5.3841).

### 2.6. Western Blotting

Mitochondrial dynamics (Pink1, Mfn2, and Drp1), inflammation markers (NLRP3, Caspase-1, and IL-1β), and proteins related to antioxidant pathways (Nrf2 and phosphorylated Nrf2) were assessed in hippocampal tissues using Western blotting. The tissues were homogenized in ice-cold RIPA lysis buffer (containing 50 mM of Tris–HCl, pH 7.4, 150 mM of NaCl, 1% NP-40, 0.25% sodium deoxycholate, 1 mM of ethylenediaminetetraacetic acid, 1 mM of phenylmethylsulfonyl fluoride, 1 mM of sodium orthovanadate, 1 mM of sodium fluoride, and a protease inhibitor cocktail) with a Dounce tissue homogenizer. The homogenate was sonicated briefly with an Omni Ruptor 4000 for about 1 s and then centrifuged at 14,000× *g* for 15 min at 4 °C to obtain the supernatant. Protein concentrations were determined using the Bradford protein assay (Bio-Rad, Hercules, CA, USA). Proteins were separated by 10–13% SDS-PAGE and transferred to a nitrocellulose membrane using a semi-dry transfer method (40 min at 25 V) (Bio-Rad, Transblot). The membranes were blocked with 10% nonfat dry milk in PBS-T overnight at 4 °C, except for Caspase-1, which was blocked with 5% bovine serum albumin. Membranes were then incubated with primary antibodies against Nrf2 (1:2000), phospho-Nrf2-S40 (1:1000), NLRP3 (1:1000), IL-1β (1:2000), Caspase-1 (1:1000), Pink1 (1:1000), Mfn2 (1:2000), and Drp1 (1:1000). Anti-β-actin (1:5000) served as the internal control. The membranes were rinsed thrice with PBS-T for a 10-minute period, probed with the corresponding secondary antibodies (diluted 1:5000) for an hour at room temperature, and then washed three times with PBS-T for a 10-minute period. The protein bands were visualized using an enhanced chemiluminescence reagent (Pierce ECL Western Blotting Substrate, Thermo-Fisher Scientific, Waltham, MA, USA). Subsequently, the relative densities of the protein bands were analyzed using the Image Lab software (version number: 6.1.0.07) (Bio-Rad, Hercules, CA, USA).

### 2.7. Determination of Malondialdehyde (MDA) and Glutathione (GSH) Levels in the Hippocampus

The levels of the lipid peroxidation marker MDA and the endogenous antioxidant GSH in hippocampal tissues were measured. Tissue samples were first weighed and homogenized with a 10% trichloroacetic acid (TCA) solution, using a volume nine times the tissue weight. The homogenates were then centrifuged at 10,000 rpm for 10 min at 4 °C, and the supernatants were separated for MDA and GSH analysis.

For MDA measurement, an equal volume of TCA (Sigma Aldrich, St. Louis, MO, USA) was mixed with the supernatant and heated for 15 min. The samples were then analyzed using a UV-2600 spectrophotometer at 535 nm with UVProbe software (version number: v2.70). MDA levels were quantified using an extinction coefficient of 1.56 × 10^−5^ M^−1^ cm^−1^.

For GSH measurement, 250 µL of the supernatant was incubated with 1000 µL of disodium phosphate and 125 µL of a reagent based on a modified Ellman’s method [33]. This reagent was prepared by dissolving 40 mg of 5,5′-dithiobis-(2-nitrobenzoic acid) (Sigma, St. Louis, MO, USA) in 100 mL of 1% sodium nitrate. GSH levels were then detected spectrophotometrically using the UV-2600 spectrophotometer at 412 nm with UVProbe software (version number: v2.70).

### 2.8. Cognitive and Behavioral Experiments

Each rat group underwent several tests to assess the impact of the 3-day liraglutide treatment on cognitive and behavioral functions. All tests were conducted under controlled conditions of temperature, humidity, and light. Sessions were recorded with a ceiling-mounted CCD camera, and data were analyzed using the EthoVision XT computerized image analysis system (Noldus Information Technology, Wageningen, The Netherlands).

An open-field test (OFT) was used to measure general locomotor activity [34,35,36]. Rats were placed in the center of an 80 × 80 cm black plexiglass arena divided into 16 squares and recorded for 5 min. The number of squares crossed by the rats during this period was monitored and analyzed, with each movement between squares counted as a “crossing”. The arena was cleaned with 70% ethanol between trials.

To assess anxiety, the elevated plus maze (EPM) test was employed [37,38]. The EPM consists of two open (50 × 10 × 40 cm) and two closed (50 × 10 × 40 cm) arms connected via a central square (10 × 10 cm), arranged in a plus configuration and elevated 50 cm above the ground. Rats were placed in the center of the maze, facing an open arm, and allowed to explore for 5 min. In the EPM test, the avoidance of open environments is indicative of a general avoidance of physiological stress conditions [39]. In this experiment, the time spent in and the number of entries into the open and closed arms were recorded and analyzed, with each entry defined as all four limbs entering an arm. During the procedure, the apparatus was cleaned with 70% ethanol between trials.

The Morris water maze (MWM) test was used to evaluate spatial learning and memory skills [40]. The MWM comprises a black circular pool with a diameter of 150 cm and a wall height of 60 cm. In this experiment, the pool was filled with water at a temperature of 25 °C ± 1 °C to a depth of 30 cm. Four starting positions were located around the periphery of the maze at equal distances, thereby dividing it into four equal quadrants. A black escape platform (10 × 10 cm) was placed 2 cm below the water surface in the center of one of the quadrants (the target quadrant). The escape platform remained in the same quadrant throughout the experiment. Abundant extra-maze cues were provided via wall posters with geometrical figures, lamps, and furniture. During the acquisition phase, each rat was subjected to two training sessions (with a 2-h resting interval between sessions) comprising four trials per day for four consecutive days. The starting position was randomly selected for each session. In each trial, the rat was released gently from the starting position into the water, with its face oriented towards the pool wall. The rats were permitted to swim for a period of 90 s to find the platform, after which they were allowed to rest on the platform for a further 30 s. Subsequently, the latency to find the platform was recorded. In the presence of an unsuccessful attempt to locate the platform within the allotted 90 s, the rat was manually guided to the platform and allowed to rest there for 30 s. In such cases, the latency to find the platform was recorded as 90 s. The objective was to evaluate the escape latency during the acquisition phase. On the fifth day of the MWM test, a probe trial phase was conducted, during which no platform was present in the pool. Each rat was allowed to swim freely for 90 s. The time spent in the target quadrant and the number of platform crossings were measured and evaluated for spatial memory retention.

### 2.9. Statistical Analysis

All statistical analyses were conducted and presented using GraphPad Prism 9 (GraphPad Software Inc., San Diego, CA, USA). Continuous data were summarized using “mean ± SD” or “median ± IQR”, depending on the normality of the distribution, which was assessed using the Shapiro–Wilk test. For the normally distributed data, group comparisons were conducted via a one-way ANOVA or independent-samples *t*-test, depending on the group number. For the data that departed from the normal distribution, a comparison was performed using the Kruskal–Wallis test or Mann–Whitney U test, depending on the group number. A post hoc analysis for multiple comparisons was performed using the Mann–Whitney U test or Tukey’s HSD test for a Kruskal–Wallis omnibus or a one-way ANOVA, respectively. A repeated-measures ANOVA was employed to analyze the escape latency data from the MWM test. A *p*-value < 0.05 was used to indicate statistical significance.

## 3. Results

### 3.1. Liraglutide Treatment Decreased Inflammation in the Hippocampal Tissues of Rats with Epilepsy

**Figure 2:**
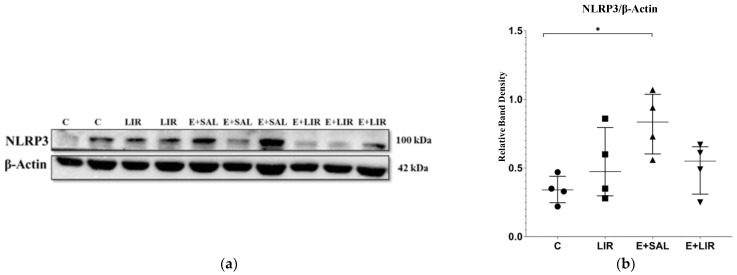

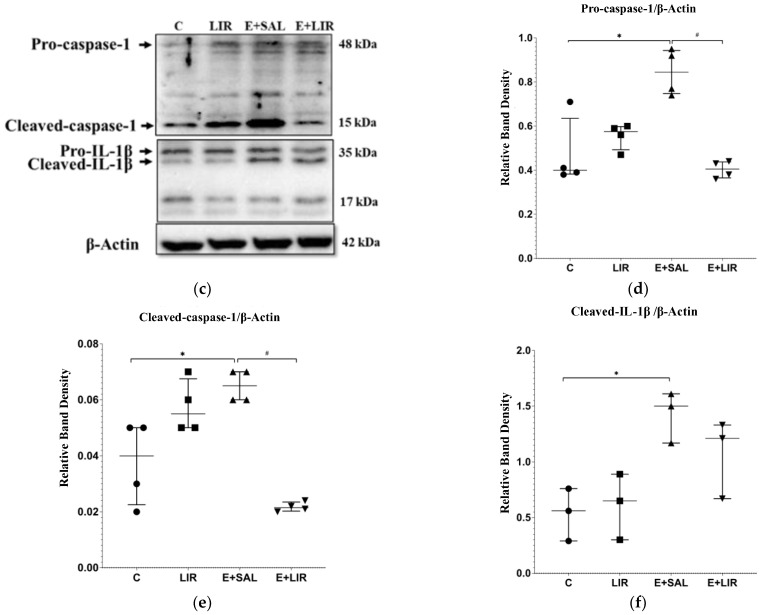
Western blotting of neuroinflammation-related proteins in hippocampal tissues of rats. (**a**–**f**) Relative expression of NLRP3, Caspase-1, and IL-1β protein bands were quantified using Image Lab. Actin was used for internal loading control. The plots show “median ± IQR” with each point corresponding to the measurement from a single animal. Comparisons of sample groups with either the control (C) group (* *p* < 0.05) or the E+SAL group (# *p* < 0.05) were performed via a Mann–Whitney U test. NLRP3, nucleotide-binding oligomerization domain-like receptor pyrin domain containing 3; IL-1β, interleukin-1β; C, control group (circles); LIR, liraglutide control group (squares); E+SAL, SE-induced group (triangles up); E+LIR, SE-induced and liraglutide-treated group (triangles down).

This study investigated whether liraglutide affects the NLRP3 inflammasome pathway in TLE because this signaling pathway is activated via mitochondria-derived ROS. The expression levels of NLRP3, IL-1β, and Caspase-1 proteins in hippocampal tissues were assessed via Western blot analysis. We observed that NLRP3 protein levels increased in SE-induced rats (Figure 2a,b). Liraglutide treatment reduced NLRP3 levels in rats with epilepsy, although no significant changes were observed (Figure 2a,b). Following NLRP3 activation, SE-induced rats had significantly higher levels of pro-Caspase-1 and cleaved-Caspase-1 than those in the control group (Figure 2c–e). Furthermore, liraglutide treatment significantly reduced the levels of both pro-Caspase-1 and cleaved-Caspase-1 in rats with TLE (Figure 2c–e). Although a slight increase in cleaved-Caspase-1 was observed in the liraglutide-treated healthy controls, this was not statistically significant. Moreover, SE-induced rats demonstrated increased cleaved-IL-1β levels (Figure 2f). A slight decreasing trend was observed in rats with epilepsy treated with liraglutide (Figure 2f).

### 3.2. Liraglutide Treatment Recruited Antioxidant Pathways in the Hippocampal Tissues of Rats with Epilepsy

**Figure 3:**
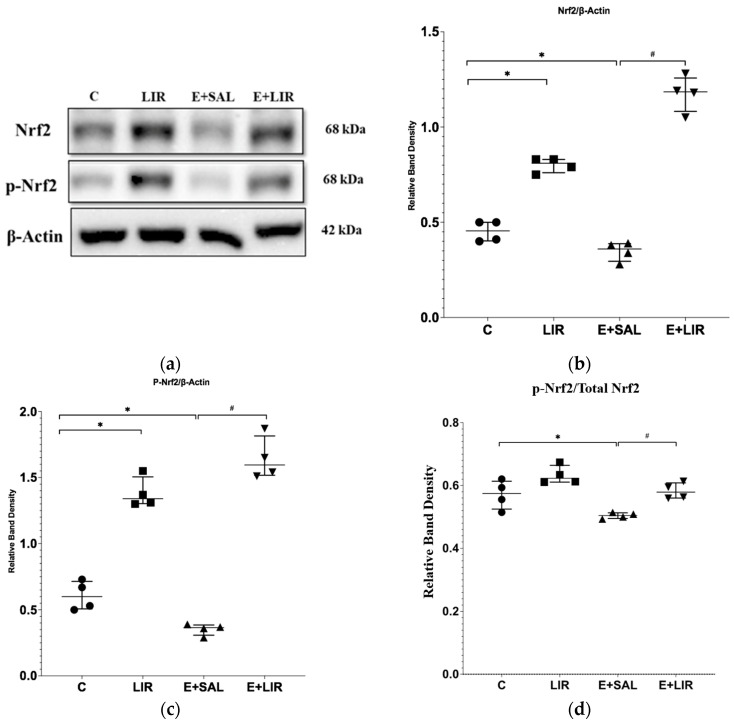
Western blotting of antioxidant pathway-related proteins in hippocampal tissues of rats. (**a**–**c**) The relative expression of Nrf2 and p-Nrf2 protein bands was quantified using Image Lab. Actin was used as an internal loading control. (**d**) The ratio of p-Nrf2 to total Nrf2. The plots show “median ± IQR” with each point corresponding to the measurement from a single animal. Comparisons of sample groups with either the control (C) group (* *p* < 0.05) or the E+SAL group (# *p* < 0.05) were performed via a Mann–Whitney U test. Nrf2, nuclear factor E2-related factor 2; p-Nrf2, phosphor-Nrf2; C, control group (circles); LIR, liraglutide control group (squares); E+SAL, SE-induced group (triangles up); E+LIR, SE-induced and liraglutide-treated group (triangles down).

Nrf2 has been reported to be one of the regulator proteins of the NLRP3 inflammasome pathway [14]. When Nrf2 is triggered via ROS and activated, it shields cells from inflammation-induced oxidative stress [16]. In this study, how liraglutide affects antioxidant pathways was examined. Western blot analyses showed that Nrf2 and p-Nrf2 protein levels were significantly lower in SE-induced rats compared to the control group whereas, liraglutide treatment led to a significant increase in these protein levels in both SE-induced and healthy control rats (Figure 3a–c). The ratio of p-Nrf2 to total Nrf2 was also calculated. The ratios indicated that p-Nrf2 levels were significantly reduced in the SE-induced group compared to the control group (Figure 3a,d). Conversely, liraglutide treatment elevated this ratio in both SE-induced and healthy control rats (Figure 3a,d).

**Figure 4:**
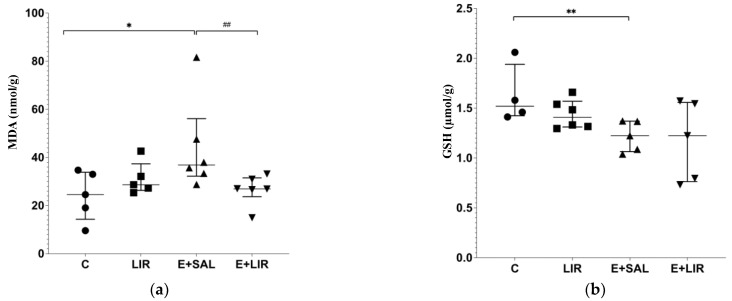
Variations in (**a**) MDA and (**b**) GSH levels in hippocampal tissues of rats. The plots show “median ± IQR” with each point corresponding to the measurement from a single animal. Comparisons of sample groups with either the control (C) group (* *p* < 0.05 and ** *p* < 0.001) or the E+SAL group (## *p* < 0.001) were performed via a Mann–Whitney U test. MDA, malondialdehyde; GSH, glutathione; C, control group (circles); LIR, liraglutide control group (squares); E+SAL, SE-induced group (triangles up); E+LIR, SE-induced and liraglutide-treated group (triangles down).

MDA and GSH levels are regarded as reliable markers of oxidative stress [41]. To determine whether liraglutide affects these markers, the levels of the lipid peroxidation marker MDA and the antioxidant enzyme GSH in hippocampal tissues were investigated using spectrophotometric analysis. SE-induced rats exhibited significantly higher MDA levels and reduced GSH levels compared to the control group (Figure 4a,b). In contrast, liraglutide reduced MDA levels in the epileptic group (Figure 4a). Altogether, these data indicate that liraglutide ameliorates the antioxidant pathways under SE-induced TLE conditions.

### 3.3. Liraglutide Treatment Changed Mitochondrial Dynamics in the Hippocampal Tissues of Rats with Epilepsy

**Figure 5:**
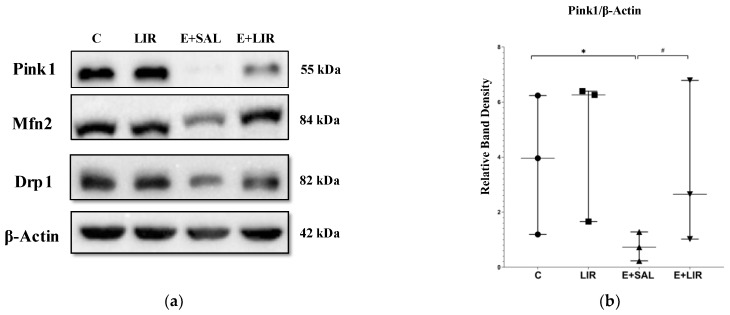

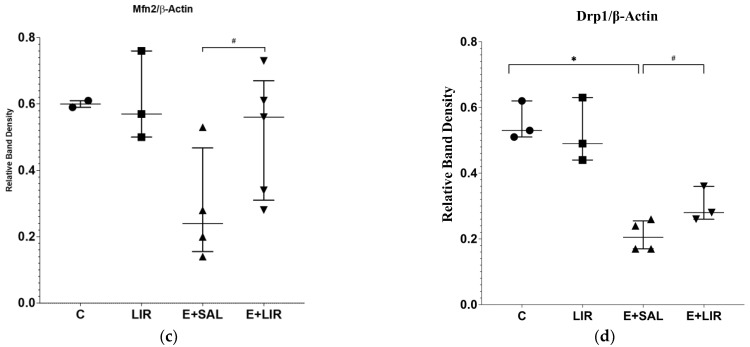
Western blotting mitochondrial dynamics-related proteins in hippocampal tissues of rats. (**a**–**d**) Relative expression of Pink1, Mfn2, and Drp1 protein bands was quantified using Image Lab. Actin was used as an internal loading control. The plots show “median ± IQR” with each point corresponding to the measurement from a single animal. Comparisons of sample groups with either the control (C) group (* *p* < 0.05) or the E+SAL group (# *p* < 0.05) were performed via a Mann–Whitney U test. Pink1, PTEN-induced kinase 1; Mfn2, mitofusin 2; Drp1, dynamin-related protein-1; C, control group (circles); LIR, liraglutide control group (squares); E+SAL, SE-induced group (triangles up); E+LIR, SE-induced and liraglutide-treated group (triangles down).

The highly dynamic organelles known as mitochondria depend on continuous fission and fusion to preserve their integrity and get rid of damaged mitochondria. Fusion enables the exchange of materials, whereas fission significantly reduces the proportion of damaged mitochondria. To maintain the quality of mitochondria, damaged mitochondria were removed via mitophagy. To evaluate how liraglutide affects mitochondrial dynamics in rats with TLE and healthy controls, mitochondrial dynamics were assessed by measuring the levels of mitophagy and mitochondrial fusion and fission using Western blotting of Pink1, Mfn2, and Drp1. Western blot analysis showed a significant reduction in Pink1 levels in SE-induced rats compared to the control group (Figure 5a,b). Moreover, liraglutide treatment increased Pink1 levels in SE-induced rats (Figure 5a,b). Furthermore, a reduction in Mfn2 levels was observed in SE-induced rats, whereas liraglutide treatment significantly increased Mfn2 levels in SE-induced rats (Figure 5a,c). Similar to Mfn2, Drp1 levels were decreased in SE-induced rats (Figure 5a,d). Liraglutide treatment restored Drp1 levels in these rats (Figure 5a,d).

### 3.4. Liraglutide’s Impact on Mitochondrial Function in PBMCs

**Figure 6:**
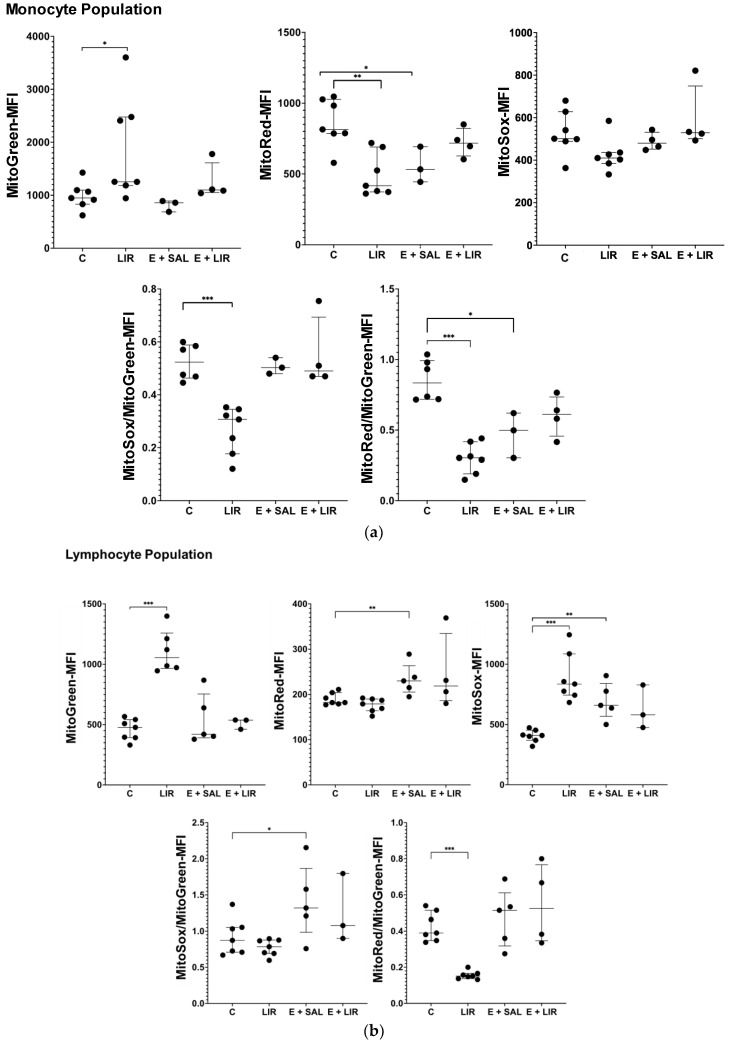
Measurement of mitochondrial function in PMBCs. Rat PBMCs were isolated using the density-gradient centrifugation method. The fluorescence intensities of MitoTracker Green, MitoTracker Red CMXRos, and MitoSox were measured in (**a**) monocyte and (**b**) lymphocyte populations. Furthermore, to estimate normalized MMP and MitoSOX, the MitoTracker Red CMXRos/ MitoTracker Green and MitoSox/MitoTracker Green ratios were calculated, respectively. The plots show “median ± IQR” with each point corresponding to the measurement from a single animal. Comparisons of sample groups with the control (C) group (* *p* < 0.05, ** *p* < 0.001, and *** *p* < 0.0001) were performed using a Mann–Whitney U test. C, control group; LIR, liraglutide control group; E+SAL, SE-induced group; E+LIR, SE-induced and liraglutide-treated group.

Growing evidence indicates that mitochondrial dysfunction is a key factor in the development of TLE [9]. PBMCs show significant mitochondrial dysfunction in several pathological conditions, including neurodegenerative diseases [18,19,42]. Therefore, this study investigated whether liraglutide affects mitochondrial function in the PBMCs of healthy and epileptic rats. For this purpose, PBMCs were stained using Mitotracker Red CMXRos, MitoSox, and MitoTracker Green FM to monitor mitochondrial membrane potential (MMP), mitochondrial superoxide production, and mitochondrial mass, respectively, using flow cytometry. Both monocyte and lymphocyte populations were analyzed separately (Figure 6). Interestingly, mitochondrial mass increased significantly in the monocyte populations of liraglutide-treated control rats (Figure 6a). In addition, we observed an increase in mitochondrial mass in the epileptic rats upon liraglutide treatment; however, it was statistically insignificant. The ratio of MitoRed (MMP) to MitoGreen (MitoMass), which indicates the levels of polarized functional mitochondria, decreased in the monocytes of epileptic rats and liraglutide-treated control rats compared to control rats. Moreover, liraglutide treatment in control rats significantly decreased MitoSox/MitoGreen ratio in this population. In the lymphocyte population (Figure 6b), the MitoGreen (MitoMass) level increased in the liraglutide control group, similar to the monocyte population. In addition, mitochondrial SOX production was enhanced; however, liraglutide treatment did not appear to cause a statistically significant difference in epileptic rats.

### 3.5. Liraglutide Treatment Reversed the Increased Locomotor Activity in Rats with Epilepsy

**Figure 7:**
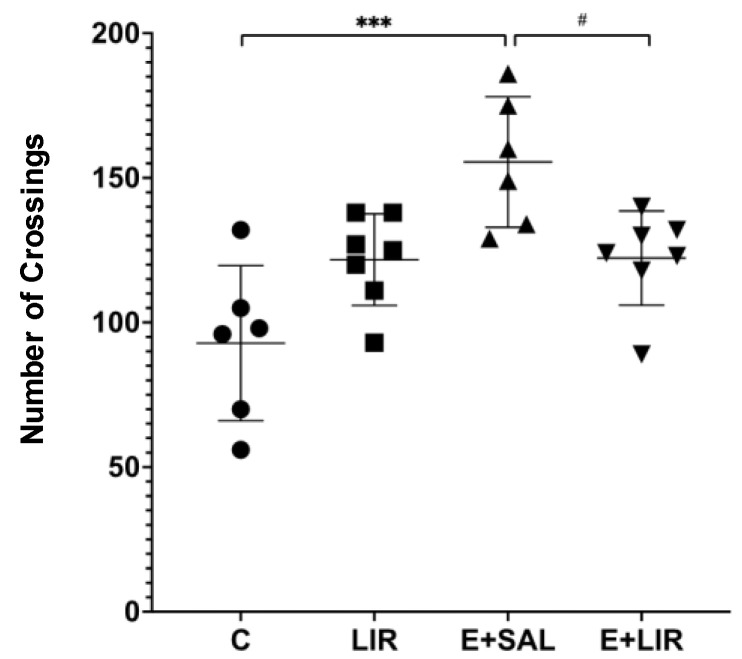
The effects of liraglutide treatment (300 µg/kg; 300 µg/1 mL/day) on locomotor activity in rats. The plots show “mean ± SD” with each point corresponding to the measurement from a single animal. A post hoc analysis for multiple comparisons was performed using Tukey’s HSD test after one-way ANOVA (*** *p* < 0.0001, # *p* < 0.05). C, control group (circles); LIR, liraglutide control group (squares); E+SAL, SE-induced group (triangles up); E+LIR, SE-induced and liraglutide-treated group (triangles down).

The OFT was conducted to assess locomotor activity in both control rats and those with epilepsy. Figure 7 shows that SE-induced rats exhibited significantly increased locomotor activity, evidenced by the increased number of crossings, and liraglutide treatment reversed this increase in locomotor activity.

### 3.6. Liraglutide Treatment Did Not Affect Anxiety-Related Behavior in Rats with Epilepsy

**Figure 8:**
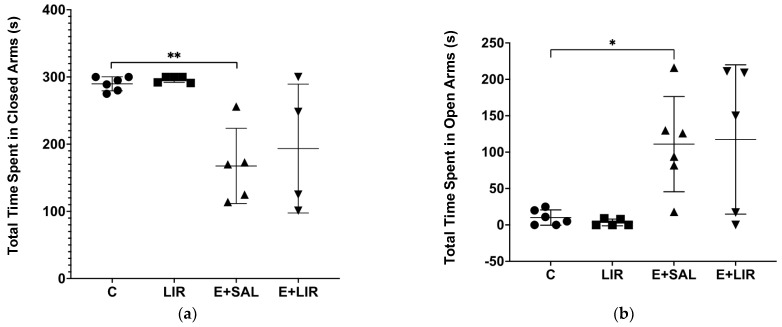
The effects of liraglutide treatment (300 µg/kg; 300 µg/1 mL/day) on anxiety-related behavior in rats. The percentage of total time spent in (**a**) closed and (**b**) open arms of the EPM. The plots show “mean ± SD” with each point corresponding to the measurement from a single animal. A post hoc analysis for multiple comparisons was performed using Tukey’s HSD test after a one-way ANOVA (* *p* < 0.05 and ** *p* < 0.001). C, control group (circles); LIR, liraglutide control group (squares); E+SAL, SE-induced group (triangles up); E+LIR, SE-induced and liraglutide-treated group (triangles down).

The EPM test was utilized to assess anxiety-related behaviors in both control and rats with epilepsy, measuring the percentage of time spent in open versus closed arms of the maze. SE-induced rats spent notably less time in the closed arms (Figure 8a) and more time in the open arms (Figure 8b) compared to controls. Consequently, liraglutide treatment did not alter the anxiety levels in epileptic animals.

### 3.7. Effects of Liraglutide Treatment on Learning and Memory in Rats with Epilepsy

**Figure 9:**
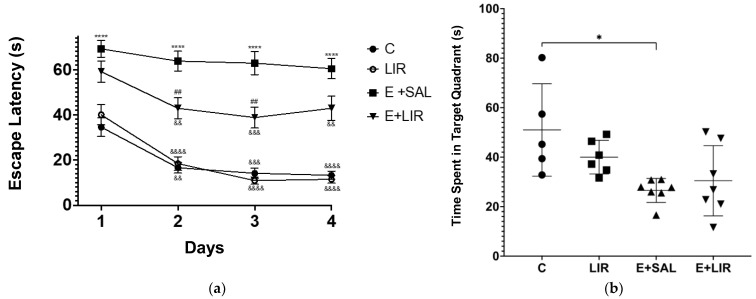
The effects of liraglutide treatment (300 µg/kg; 300 µg/1 mL/day) on learning and memory abilities in rats. (**a**) The escape latency and (**b**) time spent in the target quadrant during the MWM test. A repeated-measures ANOVA was used to analyze the escape latency data. The plots show “mean ± SD” with each point corresponding to the measurement from a single animal. A post hoc analysis for multiple comparisons was performed using Tukey’s HSD test (* *p* < 0.05, **** *p* < 0.00001 compared with the C group; ## *p* < 0.001 compared with the E+SAL group; && *p* < 0.001, &&& *p* < 0.0001, &&&& *p* < 0.00001 compared with day 1 in their group). C, control group (circles); LIR, liraglutide control group (squares); E+SAL, SE-induced group (triangles up); E+LIR, SE-induced and liraglutide-treated group (triangles down).

To investigate the impact of liraglutide treatment on spatial learning and memory, the MWM test was conducted. Figure 9a illustrates a statistically significant reduction in the mean latency to find the platform during the four training days in all groups relative to their initial performance on the initial training day. However, this trend was not observed in the saline-treated SE group. These data demonstrate that all rats, except for the E+SAL group, learned the task. Within the 4-day acquisition phase, when the E+SAL group was compared with the C group, the escape latency of SE-induced rats was significantly increased. On the other hand, liraglutide treatment decreased the time taken to find the platform for rats with epilepsy on the second and third days.

As illustrated in Figure 9b, the probe trial revealed a notable difference in the time spent in the target quadrant between the E+SAL and C groups. SE-induced rats exhibited a reduced tendency to explore the target quadrant relative to control rats. While liraglutide treatment in TLE demonstrated a slight increase in the time spent in the target quadrant, no statistically significant difference was observed between the E+LIR and E+SAL groups.

## 4. Discussion

In this study, we investigated how liraglutide affects mitochondrial dynamics and function, inflammation, antioxidant pathways, and cognitive and behavioral changes in a rat model of SE-induced TLE. We found that liraglutide may have modulatory effects on neuroinflammation and mitochondrial functions. Subsequent behavioral analysis revealed that liraglutide mitigated the increased locomotor activity often observed in epilepsy.

In the central nervous system, the neuroprotective effects of liraglutide on SE-induced TLE may stem from its antioxidant and anti-inflammatory properties, which counter inflammation, oxidative stress, and neuronal death [43,44,45]. We observed the trend that liraglutide treatment decreased the NLRP3 inflammasome and its downstream pathway, Caspase-1 and IL-1β, in SE-induced rats. Consistent with previous studies, we detected high levels of NLPR3, Caspase-1, and IL-1β in SE-induced rats compared with those in saline-treated control rats [46,47,48]. In line with our work, NLRP3 was shown to elevate the levels of Caspase-1 and IL-1β in patients with mesial TLE, contributing to inflammation in the sclerotic hippocampus [46]. A recent study on refractory TLE suggested that NLRP3 contributes to disease progression, with its inhibition potentially lessening brain damage by decreasing IL-1β expression [13]. Consistent with our findings, liraglutide reduced IL-1β and TNF-α levels in a rat model of PTZ-induced epilepsy [49], highlighting the NLRP3 inflammasome complex as a potential target for antiepileptogenic treatments. Furthermore, a significant number of studies were mainly focused on anti-inflammatory effects of GLP-1As. However, the impact of these agonists on healthy individuals or animals remains poorly understood [50,51,52,53,54]. There are many reports on side effects of GLP-1 including nausea and mood disturbances in some cases [54,55,56,57,58]. We noticed that liraglutide induced the NLRP3 and caspase-1 signaling pathways to a slight extent, even in the healthy control group, though this did not show a statistically significant difference. This response might be associated with the side effects of liraglutide doses. Moreover, like other GPCRs, the GLP-1R biased agonism may be implicated in this signaling pathway in hippocampal tissue [58]. Taken together, our findings suggest that liraglutide affects inflammation in a context-dependent manner.

In addition to inflammatory pathways, oxidative stress-related mechanisms including Nrf-2, GSH, and MDA are involved in epileptogenesis. We demonstrated that liraglutide restored Nrf-2 and p-Nrf-2 levels in rats with SE-induced epilepsy, suggesting increased Nrf2 activity. Spectrophotometric analysis of the lipid peroxidation marker MDA and the antioxidant GSH in rat hippocampi showed increased MDA and reduced GSH levels in SE-induced rats, indicating oxidative stress. Liraglutide treatment significantly lowered the MDA levels in these rats. Pilocarpine injections increased lipid peroxidation and reduced GSH levels, contributing to neuronal death in the hippocampus [59,60]. Similarly, PTZ-kindling in mice increased lipid peroxidation, which liraglutide treatment mitigated [49,61,62,63]. However, liraglutide did not increase GSH levels in the hippocampus of SE-induced rats, possibly because of GSH synthesis depending on various factors, such as amino acids and catalytic proteins [64]. Our findings align with those of previous research, suggesting that liraglutide helps restore brain oxidative balance.

Although many studies have focused on neuronal tissues, the mitochondrial dysfunction of peripheral tissues, including fibroblasts, and blood cells, including lymphocytes and platelets, has also been reported in various neurological diseases. Furthermore, recent studies have shown that mitochondrial dysfunction in peripheral blood cells could act as a biomarker for neurodegenerative diseases like Alzheimer’s and autism spectrum disorders [17,18]. To the best of our knowledge, this is the first study to investigate the impact of liraglutide on mitochondrial functions, particularly in PBMCs in the context of TLE, aside from T2DM. MitoSox levels increased in the lymphocyte population of TLE rats. Interestingly, liraglutide increased the mitochondrial mass and decreased MMP levels in both monocyte and lymphocyte populations of control rats. Hence, our study highlights mitochondrial dysfunction in the PBMCs of epileptic rats, confirming functional changes in this population [9]. Although the direct effect of GLP-1RAs and the GLP-1R expression on PBMC remains controversial, changes in the mitochondrial function of control mice might be related to the receptor expression level of these cells. Furthermore, how GLP-1 modulates cellular functions is not well defined; our study provides mechanistic insights into the actions of GLP-1. Consequently, our study confirms that liraglutide may affect the metabolic switch of peripheral blood cells.

The precise mechanism through which GLP-1RAs, such as exenatide, liraglutide, and semaglutide, exert their beneficial effects on the nervous system remains uncertain. Whether these effects occur directly through the nervous system or are the result of other metabolic changes in the body remains unclear. The expression of GLP-1Rs increased in immune-system cells and brain tissue, suggesting a direct effect of liraglutide. In addition to its direct effects, inter-organ communication, potentially via neural pathways, contributes to the indirect anti-inflammatory actions of GLP-1RAs [22,50,65,66].

The processes of mitochondrial fusion and fission, which are regulated via proteins such as Mfn2 (fusion) and Drp1 (fission), are essential for the optimal functioning of mitochondria. Mfn2, in particular, is crucial for protecting the brain from oxidative stress [67]. We demonstrated that Mfn2 and Drp1 levels were significantly reduced in SE-induced rats compared with those in the control group. Moreover, liraglutide treatment in SE-induced rats increased both Mfn2 and Drp1 levels. A recent study showed that the expression level of Mfn2 was significantly decreased in the hippocampal tissues of a rat model of lithium–pilocarpine-induced epileptic seizure [68]. Moreover, liraglutide has been reported to increase Mfn2 levels in a mouse model of Parkinson’s disease [26]. Similar to our results, Drp1 levels decreased after SE induction [69,70]. However, contradictory results have been reported in the literature [71]. Pink1 and Parkin are also crucial for eliminating damaged mitochondria and protecting against oxidative stress [72,73,74]. We showed that rats with SE-induced epilepsy exhibited notably lower Pink1 levels than controls. In rats with lithium–pilocarpine-induced epilepsy, targeting Pink1 and enhancing its expression reduced hippocampal damage and improved mitophagy [75]. Aligning with liraglutide’s mitophagy enhancement, our findings indicate that liraglutide may promote mitochondrial health by stimulating fusion, fission, and mitophagy, potentially safeguarding mitochondrial functionality.

Locomotor activity changes may be indicators of altered neurological processes. This study showed that liraglutide mitigated the increased locomotor activity due to SE. Consistent with our findings, SE-induced animals were reported to have increased locomotor activity compared with their controls [76,77,78]. Therefore, liraglutide’s capacity to diminish such hyperactivity indicates that it may have a potential role in the management of behavioral changes.

Recent research indicates that GLP-1 influences brain areas related to mood regulation [76,79]. In our study, liraglutide did not alter anxiety levels in SE-induced rats. This aligns with the findings of other studies indicating that liraglutide is not effective in animal models of depression and anxiety and in a clinical trial in which GLP-1 had no impact on anxiety in patients with panic disorder [80,81,82]. Interestingly, de Souza et al. reported that preventive liraglutide treatment, particularly when combined with levetiracetam, could reduce anxiety-like symptoms in PTZ-kindled animals [83].

Experimental models, including KA, amygdala kindling, PTZ kindling, and lithium–pilocarpine, have shown declines in spatial and contextual memory [84,85,86]. In this study, the liraglutide-treated epileptic rats exhibited increased water-maze performance compared to epileptic rats on the second and third days. Additionally, liraglutide led to a slight, but not statistically significant, increase in the time spent in the target quadrant for rats with SE-induced TLE. This suggests that liraglutide may have a positive impact on memory. Various studies with different animal models have also shown that liraglutide treatment improves memory performance in the MWM test [87,88,89].

It is important to note that our study faced several limitations. Firstly, we conducted our research on male rats. Further studies are required to determine whether the factors we investigated in the lithium–pilocarpine-induced TLE model exhibit gender-specific characteristics. Secondly, the study focused on a single TLE model, and exploring different TLE models would be beneficial for drawing more definitive conclusions. A third limitation is the relatively limited sample size used in our study. Increasing the number of animals in future studies could lead to more robust data, which would enhance the generalizability of our findings. Finally, it would be beneficial to test different liraglutide doses and treatment times. This would help better assess their potential for translation into clinical practice.

## 5. Conclusions

In conclusion, this study demonstrated that liraglutide may have neuroprotective benefits in SE-induced TLE by modulating neuroinflammation, oxidative stress, and mitochondrial function. Furthermore, it was found that liraglutide effectively mitigated the increased locomotor activity often observed in epilepsy. This research underscores the potential of GLP-1RAs as a possibly promising therapeutic strategy for TLE.

## Data Availability

The raw data supporting the conclusions of this article will be made available by the corresponding author via email.
